# Valve-Related Outcomes up to Six Years of a Next-Generation Bovine Pericardial Bioprosthesis Compared with Its Predecessor: A Propensity-Matched Analysis

**DOI:** 10.3390/medsci14030411

**Published:** 2026-07-21

**Authors:** Alessandra Francica, Fabiola Perrone, Irene Maffei, Maria Teresa Denora, Giovanni Battista Luciani

**Affiliations:** Division of Cardiac Surgery, Department of Surgery, Dentistry, Pediatrics and Gynecology, University of Verona, 37126 Verona, Italy; perronefabiola5@gmail.com (F.P.); irene.maffei@yahoo.it (I.M.); mariateresa.denora@libero.it (M.T.D.); giovanni.luciani@univr.it (G.B.L.)

**Keywords:** aortic valve replacement, pericardial valve prosthesis, bioprosthesis, Inspiris Resilia valve, Perimount Magna Ease valve, structural valve degeneration

## Abstract

**Background:** Current ESC/EACTS guidelines recommend bioprosthetic valves for aortic valve replacement (AVR) in patients > 65 years, yet their use is increasing among younger individuals. The INSPIRIS Resilia (IR) valve, a next-generation bioprosthesis, was designed to improve durability and reduce structural valve degeneration (SVD). This study compares clinical and valve-related outcomes up to six years in patients undergoing AVR with either the IR or its predecessor, the Perimount Magna Ease (PME) valve. **Methods:** Between January 2017 and December 2023, 2053 patients underwent isolated or combined AVR at a single institution (1602 PME; 451 IR). Propensity-score matching identified 224 patient pairs with comparable baseline characteristics. Outcomes were assessed over follow-up extending up to six years, focusing on cardiovascular and valve-related adverse events. Transvalvular gradients were also assessed at follow-up. **Results:** After matching, mean age was 64.2 ± 7.7 years in both groups. Freedom from cardiovascular mortality at six years was similar between PME and IR (96.6% vs. 98.2%; *p* = 0.34), as were rates of stroke, rehospitalization, prosthetic valve endocarditis, and reintervention. A total of 12 PME (5.4%) and six IR valves (2.5%) were explanted during follow-up (*p* = 0.15), with prosthetic endocarditis representing the leading cause of reintervention. Only one case of severe SVD was observed in both groups. **Conclusions:** In patients undergoing AVR with comparable baseline risk profiles, IR and PME valves demonstrated similar clinical, valve-related, and hemodynamic outcomes up to six years of follow-up. Prosthetic valve endocarditis represented the leading cause of valve failure, whereas structural valve deterioration remained rare. Longer follow-up and a greater number of valve-related events will be necessary to determine whether differences in bioprosthetic valve degeneration emerge over time.

## 1. Introduction

In line with the latest guidelines, surgical aortic valve replacement (AVR) is indicated for patients younger than 75 years with severe aortic valve disease, with 65 years considered the threshold for selecting a bioprosthetic valve in the aortic position [1,2,3,4]. Nevertheless, over the past decades, there has been a growing trend toward the use of bioprosthetic valves in patients younger than 65 years old [5]. The third-generation stented bovine pericardial prosthesis, the Carpentier-Edwards Perimount Magna Ease (PME), has shown favorable outcomes in terms of durability and safety [6,7,8], with superior performance compared with other bioprosthetic valves, especially in the first 5–7 years from implantation [9,10]. The INSPIRIS Resilia (IR) aortic prosthesis, the most recent addition to the same product line, was developed to enhance durability through innovative tissue preservation technology, with mid-term data confirming its safety and efficacy [11,12]. However, limited real-world evidence is currently available comparing these two generations of prostheses. The aim of this study was to assess and compare the valve-related outcomes of the PME and IR aortic valve prostheses over follow-up extending up to six years in contemporary clinical practice.

## 2. Methods

### 2.1. Study Population

Data of all consecutive patients undergoing isolated or combined AVR with PME or IR valves from January 2017 to December 2023 were collected prospectively at the Division of Cardiac Surgery at the Azienda Universitaria Integrata of Verona. Preoperative, intraoperative and postoperative data were collected in a dedicated and anonymized database. All echocardiographic data were retrieved from the Institutional Database of Cardiological Referring Hospitals, which were all accredited to European standards of Echocardiography. Clinical follow-up was accomplished either by querying the Electronic Clinical Chart of each patient (retrieving data from the Regional Health Database) or by interviewing the patient. The study was conducted according to the guidelines of the Declaration of Helsinki and approved by the Ethical Committee of the Azienda Universitaria Integrata of Verona (BBCCH847CESC201, approval date: 22 January 2025). The informed consent was obtained from patients involved in the study.

### 2.2. EndpPoints

The primary endpoints were valve-related outcomes, including structural valve degeneration (SVD), prosthetic valve endocarditis, and reintervention requiring prosthesis explantation. Secondary endpoints included cardiovascular mortality, stroke, and rehospitalization for cardiovascular causes. SVD and non-SVD were assessed according to the latest Valve Academic Research Consortium-3 (VARC-3) criteria [13].

Follow-up echocardiographic data were obtained from patient interviews, reports provided by referring cardiologists, and the Regional Health Information System. Echocardiographic examinations were therefore performed independently at each patient’s referring center according to routine clinical practice, without centralized image review. Because some echocardiographic variables were inconsistently reported, analyses were restricted to parameters available in at least 50% of examinations, including mean and peak transvalvular gradients, peak velocity, left ventricular ejection fraction, left ventricular end-diastolic volume, and interventricular septal thickness. Accordingly, other prosthetic valve hemodynamic parameters, such as effective orifice area, indexed effective orifice area, Doppler velocity index and acceleration time, were not consistently available and could not be reliably analyzed. Echocardiographic assessments were analyzed at discharge, 1–3 years, and 4–6 years after surgery among patients remaining at risk with an available examination. A size-stratified analysis was also performed at each time point (small: 19–21 mm; medium: 23–25 mm; large: 27–29 mm).

### 2.3. Statistical Analysis

Descriptive statistics were used to summarize the data. Categorical variables are presented as absolute values and percentages, whereas continuous variables are expressed as mean ± standard deviation (SD) or median with interquartile range (IQR), as appropriate. Categorical variables were compared using the chi-square test or Fisher’s exact test when appropriate, depending on expected cell counts. Continuous variables were tested for normality using the Shapiro–Wilk test and compared using Student’s *t*-test or Mann–Whitney U test, as appropriate. Propensity score (PS) matching was performed using the nearest-neighbor method with a caliper width of 0.2 of the standard deviation of the logit of the propensity score. Variables included in the PS model were age, sex, body surface area (BSA), and baseline left ventricular ejection fraction (LVEF). These variables were selected a priori based on their established clinical relevance and their potential association with prosthesis selection and outcomes. Given the limited sample size and the need to preserve an adequate matched cohort, the PS model was intentionally restricted to a limited number of clinically relevant covariates. A standardized mean difference (SMD) < 0.10 was considered indicative of adequate balance between groups. Because diabetes mellitus remained the only baseline variable with a clinically relevant residual imbalance after matching (SMD = 0.40), a sensitivity analysis was performed using Cox proportional hazards regression models adjusted for diabetes mellitus. Kaplan–Meier analysis with log-rank testing was used to compare freedom from major valve-related outcomes. Competing risk analysis was performed for prosthetic valve reintervention using the Fine and Gray subdistribution hazard model, considering all-cause death as a competing event. Given the extremely low incidence of structural valve degeneration, competing risk analysis was not applied to this endpoint. Predictors of prosthetic valve endocarditis were assessed in the overall study population using Cox proportional hazards regression analysis. Univariable Cox regression analyses were initially performed including clinically relevant preoperative, intraoperative, and postoperative variables. Variables showing an association with prosthetic valve endocarditis at univariable analysis (*p* < 0.05) were subsequently entered into the multivariable model. The proportional hazards assumption was assessed using Schoenfeld residuals by testing covariate-by-time interactions. Repeated-measures ANOVA was used to compare echocardiographic parameters across different time points. A two-sided *p*-value < 0.05 was considered statistically significant. Statistical analyses were performed using SPSS Version 27.0 (IBM Corp., Armonk, NY, USA) and Stata Version 18 (StataCorp, College Station, TX, USA).

## 3. Results

### 3.1. Unmatched Population

From 2017 to 2023 a total of 2053 patients underwent isolated or combined AVR: 1602 (78%) received a PME valve, and 451 (22%) patients received an IR valve. Patients receiving PME were older (mean age 72.3 ± 8.8 vs. 59.5 ± 9.6 years old; *p* < 0.001) and were more likely to be affected by multiple comorbidities (arterial hypertension, dyslipidemia, coronary artery disease, atrial fibrillation, extracardiac arteriopathy), consequently reporting higher EuroSCORE II (5.8 ± 8.3% vs. 2.7 ± 5.4%, *p* < 0.001). IR patients were more likely to be affected by bicuspid aortic valve disease, diabetes mellitus and chronic pulmonary disease. At the time of surgery, infective endocarditis was present in 85 (5.3%) patients in the PME group and 39 (8.6%) patients in the IR group. Moderate-to-aortic regurgitation was more common in IR population (34.4% vs. 52.9%; *p* < 0.001). Preoperative clinical and echocardiographic characteristics are shown in Supplemental Tables S1 and S2. Isolated AVR was the most common intervention in both cohorts (47.5% vs. 49.9%; *p* = 0.37), followed by combined CABG in the PME population and by ascending aorta replacement in the IR population. The most frequently implanted prosthetic sizes were 21, 23 and 25 mm in both groups (Supplemental Table S3). No differences were observed in hospital mortality (0.3% vs. 0.6%; *p* = 0.15) and in postoperative incidence of major complications, except for pacemaker implantation that was significantly higher in patients implanted with PME (4% vs. 1.8%; *p* < 0.001) (Supplemental Table S4). During the six years of follow-up, cardiovascular mortality was higher in the PME population (5.2% vs. 2%; *p* = 0.003). No differences were found in rates of prosthesis explant (2.4% vs. 3.5%; *p* = 0.28). Two patients in each group underwent reoperation for severe SVD. Infective prosthetic endocarditis represented the leading cause of reintervention in both cohorts (85.7% vs. 84.6%; *p* = 0.74). Conversely, a higher rate of rehospitalization was observed among patients in the IR group. Detailed rates of all follow-up events are provided in Supplemental Table S5.

### 3.2. Propensity-Score-Matched Population

After propensity-score matching, baseline characteristics were well balanced between groups. Diabetes mellitus remained the only variable with a standardized mean difference exceeding the conventional threshold (SMD = 0.40), whereas all other baseline covariates showed satisfactory balance. A total of 224 matched pairs of patients receiving a PME or IR valve were analyzed. Mean age was 64.2 ± 7.7 in both groups, which have comparable preoperative risk profiles (EUROscore II 3.2 ± 5.06% vs. 2.7 ± 4.8, *p* = 0.13; SMD 0.1). At the time of surgery, infective endocarditis was present in 18 (8.0%) patients in the PME group and 13 (5.8%) patients in the IR group. Detailed baseline variables are summarized in Table 1 and Table 2.

#### 3.2.1. Intraoperative and Postoperative Details

After matching, no differences in the intraoperative details were found. Isolated AVR was the most common operation in both groups, followed by concomitant CABG and ascending aorta replacement. In both cohorts, the most frequently implanted prostheses were the 23 and 25 mm sizes. Postoperative survival was 100% in both groups. No differences were described in major postoperative complications, with exceptions for pacemaker implantation that remained significantly more frequent in the PME group (4% vs. 0.9%; *p* = 0.002). Intraoperative and postoperative details are reported in Table 3 and Table 4.

#### 3.2.2. Follow-Up Results

**Figure 1:**
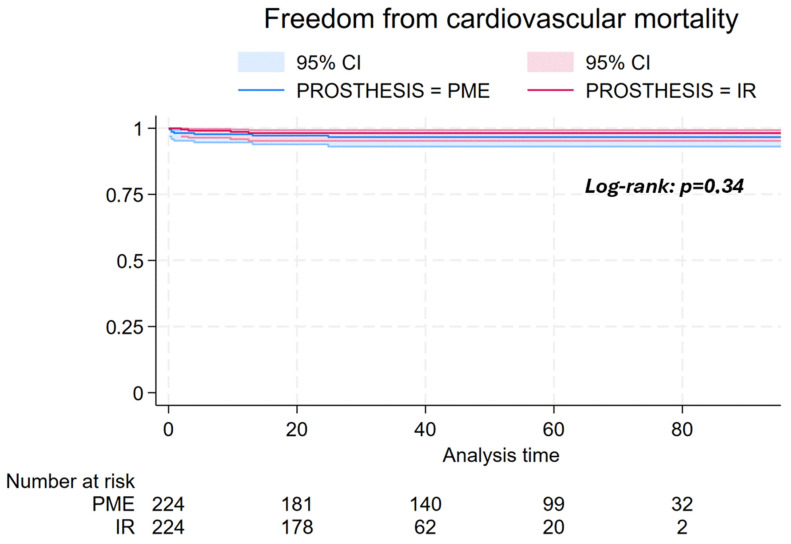
Matched populations. Kaplan–Meier estimation of freedom from cardiovascular mortality.

**Figure 2:**
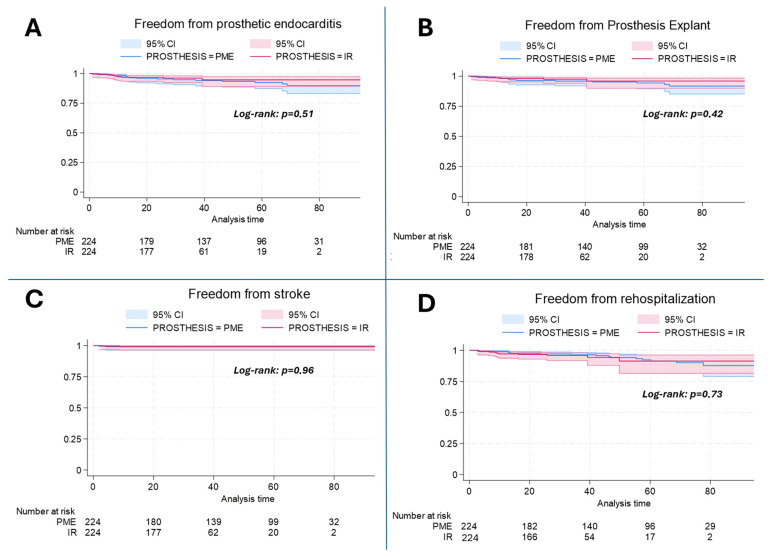
Matched populations. Kaplan–Meier estimation of Freedom from endocarditis (**A**), from prosthesis explant (**B**), from stroke (**C**), and from rehospitalization (**D**).

The median follow-up was 35.8 months (IQR 22.9–61.7), with a mean follow-up of 42.1 ± 24.8 months (range 0.13–103.6 months). The study population contributed a total of 1571.3 patient-years of follow-up. Freedom from cardiovascular mortality at six years of follow-up was comparable between the two cohorts (96.6% in the PME group vs. 98.2% in the IR group; *p* = 0.34) (Figure 1). A total of 12 PME valves (5.4%) and six IR valves (2.5%) were explanted during follow-up (*p* = 0.15), with prosthetic endocarditis representing the leading cause of reintervention (nine [75%] in the PME group vs. five [83%] in the IR group; *p* = 0.53). During follow-up, infective endocarditis occurred in 24 patients (PME: 15; IR: 9). Early infective endocarditis (≤6 months after surgery) occurred in two patients in the PME group and two in the IR group, whereas late infective endocarditis (>6 months) occurred in 13 and seven patients, respectively. The distribution of early and late events did not differ significantly between prosthesis groups (*p* = 0.57). One case of severe SVD was observed in both groups. In particular, the IR patient was 66-year-old in chronic dialysis. Two patients in the PME group experienced severe prosthesis–patient mismatch (non-SVD) requiring reoperation. Freedom from prosthesis explant, endocarditis, rehospitalization, and stroke at six years was comparable between groups (Figure 2). The cumulative incidence of reintervention was comparable between groups, with no statistically significant difference between PME and IR valves (subdistribution hazard ratio [SHR] 0.69; 95% CI 0.25–1.91; *p* = 0.47) (Supplementary Figure S1).

Because diabetes mellitus remained the only baseline variable with a clinically relevant residual imbalance after propensity-score matching (SMD = 0.40), a sensitivity analysis adjusting for diabetes mellitus was performed. Adjustment for diabetes mellitus did not materially alter the association between prosthesis type and valve-related outcomes (Supplementary Table S8), supporting the robustness of the primary findings.

In addition, a subanalysis restricted to patients undergoing isolated AVR was also performed; no statistically significant differences were observed between PME and IR groups across any of the evaluated valve-related outcomes (Supplementary Tables S9–S11). Notably, cardiopulmonary bypass and aortic cross-clamp times remained shorter in the IR group, despite the absence of concomitant procedures. This finding may be related to the presence of dry storage, which could facilitate prosthesis preparation and contribute to a more efficient surgical workflow.

#### 3.2.3. Risk Factors for Prosthetic Endocarditis at Follow-Up

Given that prosthetic valve endocarditis represented the leading cause of reintervention during follow-up in both the unmatched and matched population, a Cox proportional hazards regression analysis was performed to identify its independent predictors. Discharge aortic mean gradient (HR 1.075, 95% CI 1.026–1.126; *p* = 0.003) and cardiopulmonary bypass time (HR 1.006, 95% CI 1.002–1.010; *p* = 0.007) were independently associated with an increased risk of prosthetic valve endocarditis. Age showed a modest but significant inverse association with the outcome (HR 0.972, 95% CI 0.950–0.994; *p* = 0.013) (Supplemental Table S7). No significant association was found between prosthesis type and prosthetic valve endocarditis (HR 1.11, 95% CI 0.63–1.95; *p* = 0.717).

#### 3.2.4. Prosthetic Hemodynamic at Follow-Up

**Figure 3:**
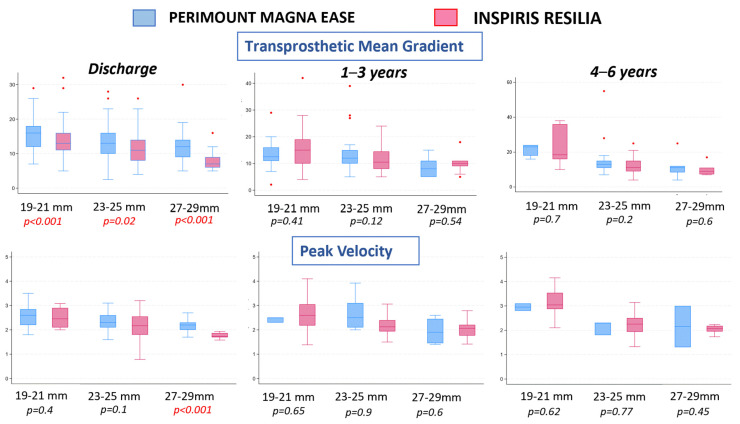
Matched population. Hemodynamic valve performance stratified by size category (small: 19–21 mm; medium: 23–25 mm; large: 27–29 mm) at different time periods (discharge, 1–3 years and 4–6 years).

Echocardiographic data at discharge were available in 208/224 (92.9%) patients in the IR group and 198/224 (88.4%) patients in the PME group. Among patients remaining at risk, echocardiographic examinations at 1–3 years were available in 94/178 (52.8%) and 59/181 (32.6%) patients in the IR and PME groups, respectively. At 4–6 years, echocardiographic data were available in 44/62 (71.0%) patients in the IR group and 53/140 (37.9%) patients in the PME group. The analysis of prosthetic hemodynamic revealed initially lower transprosthetic mean gradients at discharge in IR valves, overall (14.1 ± 5.4 mmHg vs. 11.94 ± 4.3 mmHg; *p* < 0.001), and among all size categories (Figure 3 and Supplemental Table S6). However, mean gradients became comparable between groups during follow-up (13.74 ± 8.1 mmHg vs. 12.95 ± 6.7 mmHg at 1–3 years, *p* = 0.5; 15.05 ± 5.1 mmHg vs. 14.14 ± 5.6 at 4–6 years, *p* = 0.18). Transvalvular peak velocities remained similar throughout the entire observation period (Figure 3 and Supplemental Table S6).

## 4. Discussion

This study represents one of the largest and longest propensity-matched comparisons between the PME and the IR prosthesis in contemporary surgical practice. In a matched population with a mean age of 64 years, both prostheses demonstrated comparable valve-related clinical outcomes through six years of follow-up, including cardiovascular mortality, prosthetic valve explantation, structural valve deterioration, stroke, rehospitalization for cardiovascular causes, and overall hemodynamic performance. However, interpretation of long-term hemodynamic comparisons requires caution, as echocardiographic follow-up availability decreased over time and differed between groups. Therefore, late echocardiographic findings should be considered representative of patients with available follow-up data and may have been influenced by differential follow-up completion.

Only a limited number of propensity-score-matched and comparative studies have evaluated outcomes between IR and PME valves, all with short-term follow-up not exceeding three years; these investigations consistently reported similar survival, freedom from major adverse events, and satisfactory hemodynamic performance for both valves, with only minor differences in early postoperative gradients that did not translate into clinically meaningful benefits [14,15,16,17,18]. Our findings are fully consistent with these previous reports while extending the available evidence by providing longer follow-up in a propensity-matched cohort representative of contemporary patients undergoing surgical AVR at relatively young age.

Longer follow-up results of IR valve have been reported by the COMMENCE Trial [19,20]. Comparative analysis of data from the COMMENCE trial and the PARTNER 2A contemporary AVR arm demonstrated lower five-year rates of SVD in patients receiving the IR valve (1.0% vs. 4.8%). Moreover, the use of the IR was associated with a reduced incidence of late adverse valve-related events [20]. However, it is not a direct comparison; populations start from different risk profiles and the type of bioprosthesis in the PARTNER trial is not recognized.

The rationale for comparing these two prostheses derives from their close technological relationship. The PME valve has demonstrated excellent long-term clinical performance and durability, becoming one of the most extensively implanted bovine pericardial bioprostheses worldwide, with consistently low rates of structural valve degeneration over long-term follow-up [6,7,8,9,10,11,12,13,14,15,16,17,18,19,20,21]. The IR valve was subsequently developed from the same platform with the specific aim of improving tissue preservation rather than modifying prosthesis design. RESILIA tissue incorporates stable aldehyde capping technology, which neutralizes residual free aldehyde groups within the tissue, thereby reducing calcium-binding sites and limiting mineralization. In addition, glycerol-based preservation avoids permanent glutaraldehyde storage and has been shown in preclinical studies to reduce lipid-mediated calcification and tissue mineralization compared with conventionally treated bovine pericardium [22,23]. These biological characteristics provide the mechanistic rationale for the anticipated improvement in long-term valve durability.

Our study did not demonstrate differences in structural valve deterioration between the two prostheses during the present follow-up. Only one severe SVD event occurred in each group, preventing any robust comparison of long-term valve durability. This observation should not be interpreted as evidence of equivalent durability but rather as confirmation that both prostheses provide excellent mid-term valve-related outcomes with an extremely low incidence of clinically relevant structural valve degeneration. In our cohort, the only patient developing severe SVD after IR implantation was undergoing chronic dialysis, a well-recognized condition to accelerate bioprosthetic valve degeneration through disturbances in calcium-phosphate metabolism and accelerated tissue calcification [3,9,24]. An additional feature of the IR prosthesis is its expandable stent frame, specifically developed to facilitate future transcatheter valve-in-valve procedures. As bioprosthetic valves are increasingly implanted in relatively younger patients, lifetime management strategies have become an important consideration during prosthesis selection. Nevertheless, the long-term effectiveness of valve-in-valve approaches remains uncertain, particularly with respect to prosthesis–patient mismatch, residual transvalvular gradients, coronary access, and durability of subsequent transcatheter prostheses. Whether lifetime management should preferentially rely on future valve-in-valve interventions or on more aggressive surgical strategies at the index operation, including annular or root enlargement to maximize prosthesis size, remains an area of ongoing investigation.

Interestingly, although the rationale for adopting newer bioprosthetic technologies has largely focused on delaying structural valve degeneration, our findings suggest that prosthetic valve endocarditis currently represents a more frequent cause of valve failure than SVD during mid-term follow-up. Approximately 80% of all valve explantations in our series were performed because of infective endocarditis, whereas severe structural valve deterioration remained exceptionally uncommon. This observation is consistent with the evolving epidemiology of prosthetic valve failure. While improvements in tissue preservation have substantially reduced early structural degeneration, prosthetic valve endocarditis remains one of the most devastating complications after surgical valve replacement, accounting for nearly one-third of all cases of infective endocarditis and being associated with high morbidity, mortality, and a frequent need for complex redo surgery [25,26,27,28].

Recent evidence also suggests that the clinical burden of prosthetic valve endocarditis is increasing, likely reflecting the growing number of biological valve implantations, longer patient survival, an aging and increasingly comorbid population, and the expanding use of transcatheter valve therapies and intracardiac devices [25,26,27,28].

Importantly, prosthesis type was not independently associated with prosthetic valve endocarditis in our Cox regression analysis. Although active infective endocarditis at the time of the index operation was considered, detailed microbiological data and other infection-specific risk factors were not systematically available and could not be evaluated. Instead, higher discharge transprosthetic gradients, longer cardiopulmonary bypass time, and younger age emerged as independent predictors, suggesting that the risk of prosthetic valve infection is driven predominantly by patient- and procedure-related factors rather than by prosthesis design itself.

As biological prostheses are increasingly implanted in younger patients and lifetime valve management strategies become more common, the assessment of valve performance should extend beyond durability alone. Future studies should consider prosthetic valve endocarditis as an equally relevant determinant of long-term valve-related outcomes, since freedom from reintervention may ultimately depend on both degenerative and non-degenerative mechanisms of valve failure.

### Limitations

Several limitations should be acknowledged. First, this was a retrospective observational study, and residual confounding cannot be completely excluded despite propensity-score matching. The propensity score model was intentionally restricted to clinically relevant baseline variables to balance the need for adequate confounder adjustment with the preservation of a sufficiently large, matched cohort, given the limited sample size and substantial baseline differences between groups. As a consequence, diabetes mellitus represented the only baseline variable with clinically relevant residual imbalance after matching. However, a sensitivity analysis including adjustment for diabetes mellitus yielded consistent results, supporting the robustness of the primary findings.

Second, prosthesis selection was left to surgeon preference and may therefore have introduced selection bias. Nevertheless, this reflects contemporary real-world surgical practice and enhances the external validity of our results. Although both prostheses were implanted during the same study period, the IR valve was progressively adopted in routine clinical practice following its introduction, resulting in shorter follow-up availability and fewer patients remaining at risk at later time points. This temporal implantation pattern should be considered when interpreting longitudinal echocardiographic analyses.

Third, follow-up echocardiography was performed independently at referring institutions according to routine clinical practice, without centralized image review. Consequently, some echocardiographic variables were incompletely reported, precluding a comprehensive assessment of prosthetic valve hemodynamics. Longitudinal imaging data were unavailable for all patients, and interobserver variability cannot be excluded, although this limitation is expected to have similarly affected both study groups.

Finally, the very low incidence of severe structural valve deterioration, with only one event observed in each group, precludes any definitive comparison of long-term prosthetic durability. Although this finding is reassuring from a clinical perspective, it precludes any robust comparison of structural valve degeneration between prostheses and limits the ability to draw definitive conclusions regarding comparative long-term durability. Therefore, the present study should be interpreted primarily as an assessment of clinical, valve-related, and hemodynamic outcomes.

## 5. Conclusions

In patients undergoing surgical AVR with comparable baseline risk profiles, the IR and PME bioprostheses demonstrated similar valve-related clinical and hemodynamic outcomes during six years of follow-up. While both prostheses exhibited excellent mid-term performance with an extremely low incidence of SVD, prosthetic valve endocarditis emerged as the leading cause of valve failure and reintervention in contemporary practice. These findings suggest that, as the durability of surgical bioprostheses continues to improve, future investigations should evaluate both degenerative and non-degenerative mechanisms of valve failure. Longer follow-up will be necessary to determine whether the tissue preservation technology of the IR valve ultimately translates into superior long-term durability.

## Figures and Tables

**Table 1:** Matched population. Baseline characteristics.

Baseline Characteristics	PME Population (n = 224)	IR Population (n = 224)	*p*	SMD
Age, years	64.2 (7.7)	64.2 (7.7)	0.96	0.00
Female sex	70 (31.3)	62 (27.7)	0.41	0.08
BSA, m^2^	1.91 (0.23)	1.93 (0.22)	0.37	0.08
BMI, kg/m^2^	27.3 (4.7)	26.9 (4.9)	0.35	0.08
Diabetes mellitus	10 (4.5)	40 (17.9)	<0.001	0.40
COPD	0 (0.0)	3 (1.3)	0.30	0.10
Creatinine, mg/dL	1.01 (0.78)	1.11 (1.20)	0.29	0.09
Dialysis	2 (0.9)	6 (2.7)	0.15	0.13
EuroSCORE II, %	3.5 (5.1)	2.7 (4.8)	0.13	0.10
Hypertension	148 (66.1)	141 (63.4)	0.59	0.06
Dyslipidemia	96 (42.9)	95 (42.4)	0.92	0.01
Peripheral vascular disease	21 (9.4)	25 (11.2)	0.50	0.05
Atrial fibrillation			0.92	0.06
Paroxysmal	17 (7.6)	17 (7.6)		
Persistent	14 (6.3)	12 (5.4)		
Pacemaker	10 (4.5)	3 (1.3)	0.08	0.13
Prior cardiac surgery	15 (6.7)	19 (8.5)	0.47	0.07
Prior AVR	10 (4.5)	9 (4.0)	0.81	0.02
Coronary artery disease	52 (23.2)	58 (25.9)	0.51	0.06
Endocarditis	18 (8.0)	13 (5.8)	0.35	0.07
Aortic dissection	8 (3.6)	5 (2.2)	0.40	0.08
Bicuspid aortic valve disease	53 (23.7)	50 (22.3)	0.74	0.03

AVR: aortic valve replacement; BSA: body surface area; BMI: body mass index; COPD: chronic obstructive pulmonary disease; IR: INSPIRIS RESILIA bioprosthetic valve; PME: Perimount Magna Ease bioprosthetic valve; SMD: standardized mean difference.

**Table 2:** Matched population. Preoperative echocardiographic details.

Baseline Echocardiography	PME Population (n = 224)	IR Population (n = 224)	*p*
LVEF, %	55.8 (11.1)	56.9 (10.7)	0.29
End-diastolic volume, mL	152.5 (66.9)	133.2 (41.7)	0.02
End-systolic volume, mL	81.5 (50.1)	59.9 (30.2)	0.03
Interventricular septal thickness, mm	13.1 (3.5)	13.2 (2.1)	0.69
Moderate-to-severe aortic stenosis	146 (65.2)	144 (64.3)	0.84
Moderate-to-severe aortic regurgitation	80 (35.7)	78 (34.8)	0.84
Peak transvalvular gradient, mmHg	62.6 (29.8)	62.1 (29.9)	0.91
Mean transvalvular gradient, mmHg	41.5 (17.8)	42.5 (15.2)	0.56
Peak velocity (Vmax), m/s	3.8 (1.1)	3.9 (0.8)	0.91

IR: INSPIRIS RESILIA bioprosthetic valve; PME: Perimount Magna Ease bioprosthetic valve; LVEF: left ventricular ejection fraction.

**Table 3:** Matched population. Intraoperative details.

Intraoperative Details	PME Population (n = 224)	IR Population (n = 224)	*p*
**Aortic prosthesis size**			0.69
19 mm	16 (7.2)	18 (8.1)	
21 mm	45 (20.2)	54 (24.1)	
23 mm	81 (36.2)	77 (34.4)	
25 mm	52 (23.2)	54 (24.1)	
27 mm	25 (11.2)	16 (7.1)	
29 mm	4 (1.8)	4 (1.8)	
Isolated AVR	106 (47.3)	120 (53.6)	0.17
**Concomitant procedures**			
Mitral valve surgery	16 (7.1)	12 (5.4)	0.43
Root replacement	30 (13.4)	6 (2.7)	<0.001
Ascending aorta replacement	31 (13.8)	36 (16.1)	0.51
CABG	35 (15.6)	33 (14.7)	0.79
Other	39 (17.4)	39 (17.4)	1.00
Cardiopulmonary bypass time, min	125.1 (59.9)	110.9 (46.7)	0.007
Aortic cross-clamp time, min	93.5 (34.7)	86.9 (34.4)	0.024
Intra-aortic balloon pump support	6 (2.7)	4 (1.8)	0.52
ECMO support	1 (0.4)	2 (0.9)	0.56
Procedural mortality	0 (0.0)	0 (0.0)	—

AVR: aortic valve replacement; CABG: coronary artery bypass grafting; ECMO: extracorporeal membrane oxygenation; IR: INSPIRIS RESILIA bioprosthetic valve; PME: Perimount Magna Ease bioprosthetic valve.

**Table 4:** Matched population. Postoperative details.

Postoperative Complications	PME Population (n = 224)	IR Population (n = 224)	*p*
In-hospital mortality	0 (0.0)	0 (0.0)	—
Length of hospital stay, days	9.2 (6.7)	8.1 (7.9)	0.14
Surgical revision for bleeding	6 (2.7)	4 (1.8)	0.53
Perioperative acute myocardial infarction	1 (0.4)	4 (1.8)	0.31
**Atrial fibrillation**			0.61
Paroxysmal	57 (25.4)	66 (29.5)	
Persistent	3 (1.3)	3 (1.3)	
New permanent pacemaker implantation	9 (4.0)	2 (0.9)	0.002
**Stroke**			0.08
Transient ischemic attack (TIA)	3 (1.3)	1 (0.4)	
Minor stroke	3 (1.3)	0 (0.0)	
Major stroke	5 (2.2)	1 (0.4)	
Dialysis	1 (0.4)	1 (0.4)	1.00

IR: INSPIRIS RESILIA bioprosthetic valve; PME: Perimount Magna Ease bioprosthetic valve; TIA: transient ischemic attack.

## Data Availability

The original contributions presented in this study are included in the article/Supplementary Materials. Further inquiries can be directed to the corresponding author.

## Supplementary Materials

The following supporting information can be downloaded at https://www.mdpi.com/article/10.3390/medsci14030411/s1, Table S1: Unmatched population. Preoperative charatcteristics; Table S2: Unmatched population. Preoperative echocardiographic details; Table S3: Unmatched population. Intraoperative details; Table S4: Unmatched population. Postoperative complications; Table S5: Unmatched population. Follow-up events; Table S6: Matched population. Echocardiographic data stratified by size-category and time-period.; Table S7: Cox regression analysis of predictors of prosthetic endocarditis at follow-up; Table S8: Sensitivity analysis adjusting for diabetes mellitus in the propensity-matched cohort; Table S9: Sub analysis of Isolated AVR group. Preoperative details; Table S10: Sub analysis of Isolated AVR group. Intra and Postoperative details; Table S11: Subanalysis of Isolated AVR group. Kaplan–Meier Estimates of Freedom from Clinical Events. Figure S1: Cumulative incidence function (CIF) of prosthetic valve reintervention in PME and IR groups, with death as a competing risk (Fine and Gray model). No significant difference was observed between groups.

## Abbreviations

AVR	Aortic valve replacement
BMI	Body mass index
BSA	Body surface area
CABG	Coronary artery bypass grafting
CI	Confidence interval
COPD	Chronic obstructive pulmonary disease
ECMO	Extracorporeal membrane oxygenation
EO	Early onset
EACTS	European Association for Cardio-Thoracic Surgery
ESC	European Society of Cardiology
HR	Hazard ratio
IABP	Intra-aortic balloon pump
IQR	Interquartile range
IR	INSPIRIS RESILIA bioprosthetic valve
LVEF	Left ventricular ejection fraction
LO	Late onset
NYHA	New York Heart Association
PME	Perimount Magna Ease bioprosthetic valve
PPM	Prosthesis–patient mismatch
PS	Propensity score
PVE	Prosthetic valve endocarditis
SD	Standard deviation
SMD	Standardized mean difference
SVD	Structural valve degeneration
TIA	Transient ischemic attack
VARC-3	Valve Academic Research Consortium-3
