# Supplementary material for: Valve-Related Outcomes up to Six Years of a Next-Generation Bovine Pericardial Bioprosthesis Compared with Its Predecessor: A Propensity-Matched Analysis
Source: Med Sci (Basel). 2026 Jul 21;14(3):411. doi: 10.3390/medsci14030411 (PMC13413634; doi:10.3390/medsci14030411)
Supplement: Supplementary file 1 [file medsci-14-00411-s001.zip › medsci-4362297-supplementary.pdf]

Supplemental Table S1. Unmatched population. Preoperative charatcteristics.

Baseline Characteristics	PME Population (n = 1602)	IR Population (n = 451)	P	SMD
Age, years	72.3 (8.8)	59.6 (9.6)	<0.001	1.30
Female sex	527 (32.9)	119 (26.4)	0.009	0.13
BSA, m^2	1.9 (1.4)	1.9 (0.3)	0.52	0.00
BMI, kg/m^2	27.2 (4.7)	26.7 (4.8)	0.06	0.11
Diabetes mellitus	92 (5.7)	58 (12.8)	<0.001	0.20
COPD	113 (7.1)	55 (12.1)	<0.001	0.17
Creatinine, mg/dL	1.11 (2.14)	1.07 (1.18)	0.74	0.02
Dialysis	178 (11.1)	29 (6.4)	<0.001	0.18
EuroSCORE II, %	5.8 (8.3)	2.7 (5.4)	<0.001	0.44
Hypertension	1176 (73.4)	253 (56.2)	<0.001	0.36
Dyslipidemia	809 (50.5)	168 (37.3)	<0.001	0.26
Peripheral vascular disease	265 (16.4)	41 (9.2)	<0.001	0.21
Atrial fibrillation			<0.001	0.40
Paroxysmal	169 (89.4)	20 (4.4)		
Persistent	178 (11.1)	29 (6.4)		
Pacemaker	88 (5.5)	8 (1.8)	<0.001	0.23
Prior cardiac surgery	82 (5.1)	47 (10.5)	<0.001	0.19
Prior AVR	49 (3.1)	25 (5.6)	0.012	0.12
Coronary artery disease	551 (34.4)	87 (19.3)	<0.001	0.34
Endocarditis	85 (5.3)	39 (8.6)	0.009	0.13

Aortic dissection	55 (3.4)	18 (4.0)	0.56	0.03
Bicuspid aortic valve disease	189 (11.3)	220 (48.8)	<0.001	0.88

AVR: aortic valve replacement; BSA: body surface area; BMI: body mass index; CABG: coronary artery bypass grafting; COPD: chronic obstructive pulmonary disease; IR: INSPIRIS RESILIA bioprosthetic valve; PME: Perimount Magna Ease bioprosthetic valve; LVEF: left ventricular ejection fraction; PS: propensity score; SMD: standardized mean difference; SD: standard deviation; SVD: structural valve degeneration.

Supplemental Table S2. Unmatched population. Preoperative echocardiographic details.

**Baseline Echocardiography**	**PME Overall Population (n = 1,602)**	**IR Overall Population (n = 451)**	**P**
LVEF, %	56.8 (9.9)	57.3 (9.9)	0.75
End-diastolic volume, mL	141.4 (76.6)	143.3 (58.4)	0.99
End-systolic volume, mL	67.6 (42.1)	67.7 (46.4)	0.42
Interventricular septal thickness, mm	13.4 (2.1)	12.8 (2.2)	<0.001
**Aortic stenosis**			0.61
Moderate	134 (8.4)	42 (9.3)	
Severe	905 (56.5)	256 (56.8)	
**Aortic regurgitation**			<0.001
Moderate	289 (18.0)	98 (21.8)	
Severe	262 (16.4)	141 (31.3)	
Peak transvalvular gradient, mmHg	67.9 (24.6)	58.9 (32.9)	<0.001
Mean transvalvular gradient, mmHg	42.3 (15.8)	41.7 (17.1)	0.60
Peak velocity (Vmax), m/s	5.9 (2.5)	3.8 (1.8)	0.44

AVR: aortic valve replacement; IR: INSPIRIS RESILIA bioprosthetic valve; PME: Perimount Magna Ease bioprosthetic valve; LVEF: left ventricular ejection fraction; SD: standard deviation

Supplemental Table S3. Unmatched population. Intraoperative details.

Intraoperative Details	PME Overall Population (n = 1,602)	IR Overall Population (n = 451)	P
**Aortic prosthesis size**			0.009
19 mm	110 (6.9)	36 (8.0)	
21 mm	423 (26.4)	97 (21.6)	
23 mm	577 (36.0)	147 (32.7)	
25 mm	364 (22.7)	112 (24.9)	
27 mm	105 (6.6)	49 (10.9)	
29 mm	22 (1.4)	10 (2.2)	
Isolated AVR	761 (47.5)	225 (49.9)	0.37
**Concomitant procedures**			
Mitral valve surgery	79 (4.9)	28 (6.2)	0.28
Root replacement	134 (8.4)	20 (4.4)	0.005
Ascending aorta replacement	209 (13.0)	89 (19.7)	<0.001
CABG	335 (20.9)	49 (10.9)	<0.001
Other	84 (5.2)	40 (8.9)	0.004
Cardiopulmonary bypass time, min	122.2 (55.8)	110.3 (46.5)	<0.001
Aortic cross-clamp time, min	90.9 (36.0)	85.7 (32.0)	0.008
Postoperative intra-aortic balloon pump support	54 (3.4)	10 (2.2)	0.21

Postoperative ECMO support	14 (0.9)	2 (0.4)	0.36
Procedural mortality	5 (0.3)	1 (0.2)	0.75

AVR: aortic valve replacement; CABG: coronary artery bypass grafting; ECMO: extracorporeal membrane oxygenation; IABP: intra-aortic balloon pump; IR: INSPIRIS RESILIA bioprosthetic valve; PME: Perimount Magna Ease bioprosthetic valve; SD: standard deviation

Supplemental Table S4. Unmatched population. Postoperative complications.

**Postoperative Complications**	**PME Overall Population (n = 1,602)**	**IR Overall Population (n = 451)**	**P**
Length of hospital stay, days	10.9 (24.1)	12.0 (37.9)	0.44
In-hospital mortality	5 (0.3)	3 (0.6)	0.15
Surgical revision for bleeding	50 (3.1)	11 (2.4)	0.46
Perioperative acute myocardial infarction	9 (0.6)	5 (1.2)	0.03
**Atrial fibrillation**			0.14
Paroxysmal	489 (30.5)	117 (26.0)	
Persistent	12 (0.7)	5 (1.1)	
New permanent pacemaker implantation	64 (4.0)	8 (1.8)	<0.001
**Stroke**			0.11
Transient ischemic attack (TIA)	23 (1.4)	1 (0.2)	
Minor stroke	18 (1.1)	3 (0.7)	
Major stroke	18 (1.1)	3 (0.7)	
Dialysis	43 (2.7)	12 (2.7)	0.16

IR: INSPIRIS RESILIA bioprosthetic valve; PME: Perimount Magna Ease bioprosthetic valve; TIA: transient ischemic attack; SD: standard deviation

Supplemental Table S5. Unmatched population. Follow-up events.

Follow-up Events	PME Overall Population (n = 1,602)	IR Overall Population (n = 451)	P
Cardiovascular mortality	84 (5.2)	9 (2.0)	0.003
Prosthetic valve endocarditis	60 (3.7)	16 (3.5)	0.84
Stroke	22 (1.4)	4 (0.9)	0.42
Prosthesis explantation	38 (2.4)	16 (3.5)	0.28
**Cause of prosthesis explantation***			
Severe SVD	2 (5.3)	2 (12.5)	0.74
Endocarditis	32 (84.2)	13 (81.3)	
Non-SVD	4 (10.5)	1 (6.2)	
Rehospitalization for cardiovascular causes	69 (4.3)	43 (9.5)	<0.001

R: INSPIRIS RESILIA bioprosthetic valve; PME: Perimount Magna Ease bioprosthetic valve; SVD: structural valve degeneration; non-SVD: non-structural valve deterioration; n/N: number of events/number of patients; SD: standard deviation

*Percentages calculated among patients undergoing prosthesis explantation (PME: n = 38; IR: n = 16).

Supplemental Table S6. Matched population. Echocardiographic data stratified by size-category and time-period.

Echocardiographic data	Discharge (Mean ± SD)			1-3 years (Mean ± SD)			4 - 6 years (Mean ± SD)		
**All prosthesis size**	**PME**	**IR**	**p-value**	**PME**	**IR**	**p-value**	**PME**	**IR**	**p-value**
LVEDV (ml)	126.83 ± 59.3	119.4 ± 38.8	0.02	115.61 ± 40	109.94 ± 30.3	0.45	106.39 ± 29.6	94.11 ± 33.6	0.19
LVEF (%)	54.34 ± 10	54.93 ± 9.9	0.56	58.08 ± 10.2	59.87 ± 6.1	0.21	57.24 ± 9.2	59.61 ± 5.6	0.18
IVS (mm)	12.72 ± 1.9	12.65 ± 1.7	0.77	11.60 ± 1.8	12.05 ± 1.7	0.24	11.89 ± 1.6	12.39 ± 1.6	0.22
Peak gradient (mmHg)	28.69 ± 65.2	17.61 ± 7.7	0.17	20.19 ± 9	22.73 ± 11.7	0.22	24.37 ± 8.5	24.64 ± 11.7	0.92
Mean gradient (mmHg)	14.11 ± 5.4	11.94 ± 4.3	<.001	13.74 ± 8.1	12.95 ± 6.7	0.56	15.05 ± 5.1	14.14 ± 5.6	0.18
Vmax (m/s)	2.47 ± 1	2.05 ± 0.4	0.002	2.37 ± 0.5	2.37 ± 0.7	0.98	2.37 ± 0.6	2.39 ± 0.6	0.89
**Size 19-21mm**									
LVEDV (ml)	96.14 ± 30.7	96.77 ± 30.3	0.93	99.90 ± 54.7	101.11 ± 35.5	0.95	80.42 ± 10.2	110	0.06
LVEF (%)	57.13 ± 8.2	57.32 ± 9.8	0.90	60.90 ± 9.4	59.83 ± 6.9	0.71	59.50 ± 5.8	59.33 ± 4	0.96
IVS (mm)	12.64 ± 1.8	12.15 ± 1.3	0.24	12.75 ± 2.1	12.71 ± 1.5	0.96	11.86 ± 1.3	10.33 ± 1.1	0.12
Peak gradient (mmHg)	27.93 ± 10.7	20.06 ± 9.9	0.013	20.10 ± 8.6	29.81 ± 17.1	0.11	32.80 ± 9.1	36.67 ± 5.5	0.51
Mean gradient (mmHg)	16.32 ± 6.2	12.87 ± 4.3	<.001	15.90 ± 4.9	16.91 ± 10	0.76	19.14 ± 4.5	19.33 ± 2.5	0.94
Vmax (m/s)	2.59 ± 0.5	2.18 ± 0.5	0.009	2.53 ± 0.3	2.62 ± 0.7	0.71	2.83 ± 0.4	3.06 ± 0.3	0.39
**Size 23-25mm**									
LVEDV (ml)	136.68 ± 64.9	131.33 ± 37.1	0.42	118.16 ± 34.8	111.72 ± 28.2	0.43	111.08 ± 31.1	87.07 ± 33.5	0.04
LVEF (%)	53.48 ± 10.3	54.15 ± 9.4	0.61	57.57 ± 9.5	59.79 ± 6.2	0.18	57.34 ± 9.4	59.40 ± 5.9	0.31
IVS (mm)	12.68 ± 1.8	12.89 ± 1.8	0.54	11.41 ± 1.7	11.90 ± 1.9	0.28	11.86 ± 1.7	12.62 ± 1.5	0.12
Peak gradient (mmHg)	31.05 ± 84.7	18.46 ± 7.4	0.39	21.04 ± 9.2	21.43 ± 9	0.85	21.35 ± 6.6	24.52 ± 11.9	0.27
Mean gradient (mmHg)	13.49 ± 4.6	12.01 ± 4.2	0.015	13.45 ± 9.1	12.15 ± 4.8	0.39	13.85 ± 4.9	12.73 ± 6.1	0.50
Vmax (m/s)	2.48 ± 1.3	2.11 ± 0.4	0.10	2.38 ± 0.6	2.37 ± 0.7	0.97	2.2 ± 0.6	2.37 ± 0.6	0.31
**Size 27-29mm**									
LVEDV (ml)	151.18 ± 61.4	119.18 ± 44.4	0.15	138.83 ± 25.6	118.71 ± 33.5	0.41	124.68 ± 17	121.81 ± 23.1	0.84
LVEF (%)	51.65 ± 11.7	53.26 ± 10.3	0.64	53.67 ± 22.2	60.67 ± 2.4	0.44	50.75 ± 13.9	62 ± 3.6	0.24
IVS (mm)	13.22 ± 2.8	12.83 ± 1.8	0.70	10.33 ± 0.6	11.5 ± 1.1	0.13	12.5 ± 0.7	12.75 ± 1.5	0.81
Peak gradient (mmHg)	19.38 ± 7.4	13.07 ± 3.4	0.004	11.33 ± 0.6	15.71 ± 4.6	0.15	25.05 ± 6	16.36 ± 4.9	0.09
Mean gradient (mmHg)	11.68 ± 5.6	7.8 ± 2	0.015	9 ± 3.5	8.43 ± 2.1	0.75	14.33 ± 3.2	10.10 ± 2.8	0.16
Vmax (m/s)	2.17 ± 0.4	1.79 ± 0.2	0.002	1.79 ± 0.2	1.87 ± 0.3	0.73	2.48 ± 0.3	2.0 ± 0.3	0.08

R: INSPIRIS RESILIA bioprosthetic valve; PME: Perimount Magna Ease bioprosthetic valve; LVEDV: left ventricular end-diastolic volume; LVEF: left ventricular ejection fraction; IVS: interventricular septum thickness; Vmax: peak aortic jet velocity; SD: standard deviation

Supplementary Table S7. Cox regression analysis of predictors of prosthetic endocarditis at follow-up.

Variable	Univ. HR (IC 95%)	p	Multiv. HR (IC 95%)	p
Discharge Aortic Mean Gradient (mmHg)	1.073 (1.024–1.125)	0.003	1.075 (1.026–1.126)	0.003
Age (years)	0.972 (0.950–0.995)	0.016	0.972 (0.950–0.994)	0.013
CEC time (min)	1.005 (1.001–1.010)	0.024	1.006 (1.002–1.010)	0.007
AVR + MVR	2.047 (0.858–4.887)	0.107	—	—

HR: hazard ratio; CI: confidence interval; CEC: cardiopulmonary bypass; AVR: aortic valve replacement; MVR: mitral valve replacement.

Supplementary Table S8. Sensitivity analysis adjusting for diabetes mellitus in the propensity-matched cohort.

Outcome	Adjusted HR for IR vs PME	95% CI	p-value
Cardiovascular mortality	0.56	0.16–1.95	0.360
Prosthetic valve explantation	0.59	0.21–1.68	0.321
Prosthetic valve endocarditis	0.77	0.32–1.82	0.547
Stroke	1.11	0.16–7.85	0.920
Rehospitalization for cardiovascular causes	1.12	0.47–2.66	0.791

HR: hazard ratio; IR: INSPIRIS RESILIA bioprosthetic valve; PME: Perimount Magna Ease bioprosthetic valve; CI: confidence interval.

Supplementary Table S9. Sub analysis of Isolated AVR group. Preoperative details.

Baseline Characteristics	PME n=106	IR n=120	p
Age, years	64.0 ± 7.9	64.2 ± 8.0	0.901
Female gender	38 (35.8)	33 (27.5)	0.177
BSA, m^2	1.90 ± 0.24	1.92 ± 0.21	0.473
BMI, kg/m^2	27.6 ± 4.7	26.9 ± 4.7	0.292
COPD	7 (6.6)	23 (19.2)	0.005
Peripheral vascular disease	15 (14.2)	11 (9.4)	0.270
Hypertension	69 (65.1)	48 (61.5)	0.620
Dyslipidaemia	48 (45.3)	54 (45.0)	0.966
Atrial fibrillation – Paroxysmal	9 (8.5)	8 (6.7)	
Atrial fibrillation – Persistent	3 (2.8)	5 (4.2)	0.766
Pacemaker	4 (3.8)	1 (0.8)	0.134
Coronary artery disease	12 (11.3)	12 (10.0)	0.748
Prior AVR	4 (3.8)	5 (4.2)	0.880
Endocarditis	10 (9.4)	8 (6.7)	0.443
Aortic dissection	0 (0)	1 (0.8)	0.346
Bicuspid aortic valve disease	25 (23.6)	29 (37.2)	0.045
Moderate–severe aortic regurgitation	30 (28.3)	42 (38.9)	0.047
Moderate–severe aortic stenosis	77 (72.6)	84 (77.8)	0.303

Supplementary Table S10. Sub analysis of Isolated AVR group. Intra and Postoperative details.

**Intraoperarive details**	**PME (n=106)**	**IR (n=120)**	**p**
Cardiopulmonary bypass time, min	96.35 ± 25.35	87.47 ± 26.54	0.012
Cross clamp time, min	73.51 ± 18.86	68.25 ± 20.52	0.050
Intra-aortic balloon pump	0 (0.0%)	1 (0.8%)	0.346
ECMO postop	2 (1.9%)	2 (1.7%)	0.634
Procedural mortality	0 (0%)	0 (0%)	—
**Postoperative details**			
In-hospital stay, days	8.82 ± 6.66	7.67 ± 7.99	0.251
In-hospital mortality	0 (0%)	0 (0%)	—
Surgical revision for bleeding	1 (0.9%)	3 (2.5%)	0.371
Perioperative acute myocardial ischemia	0 (0%)	2 (1.7%)	0.407
Atrial fibrillation (total)	23 (21.7%)	31 (26.1%)	0.189
└ Paroxysmal	21	31	—
└ Persistent	2	0	—
New pacemaker implantation	0 (0%)	1 (0.8%)	1.0
Stroke (any)	4 (3.8%)	1 (0.8%)	0.431
└ Minor	3	1	—
└ Major	1	0	—
TIA	0	0	—
Dialysis	0	0	—
CVVH	2 (1.9%)	3 (2.5%)	0.634

Supplementary Table S11. Subanalysis of Isolated AVR group. Kaplan–Meier Estimates of Freedom from Clinical Events.

Outcome	PME, mean ± SE	PME 95% CI	IR, mean ± SE	IR 95% CI	Log-rank p-value
Freedom from cardiovascular death	99.78 ± 1.70	96.45–103.11	95.92 ± 1.07	93.81–98.02	0.498
Freedom from prosthesis explant	95.09 ± 2.60	89.99–100.18	92.67 ± 2.19	88.38–96.96	0.470
Freedom from endocarditis	93.79 ± 2.78	88.33–99.24	92.77 ± 1.86	89.12–96.43	0.343
Freedom from stroke	101.78 ± 0.99	99.85–103.71	96.65 ± 0.80	95.08–98.21	0.910
Freedom from rehospitalization	92.34 ± 2.88	86.70–97.98	93.28 ± 1.83	89.70–96.86	0.390

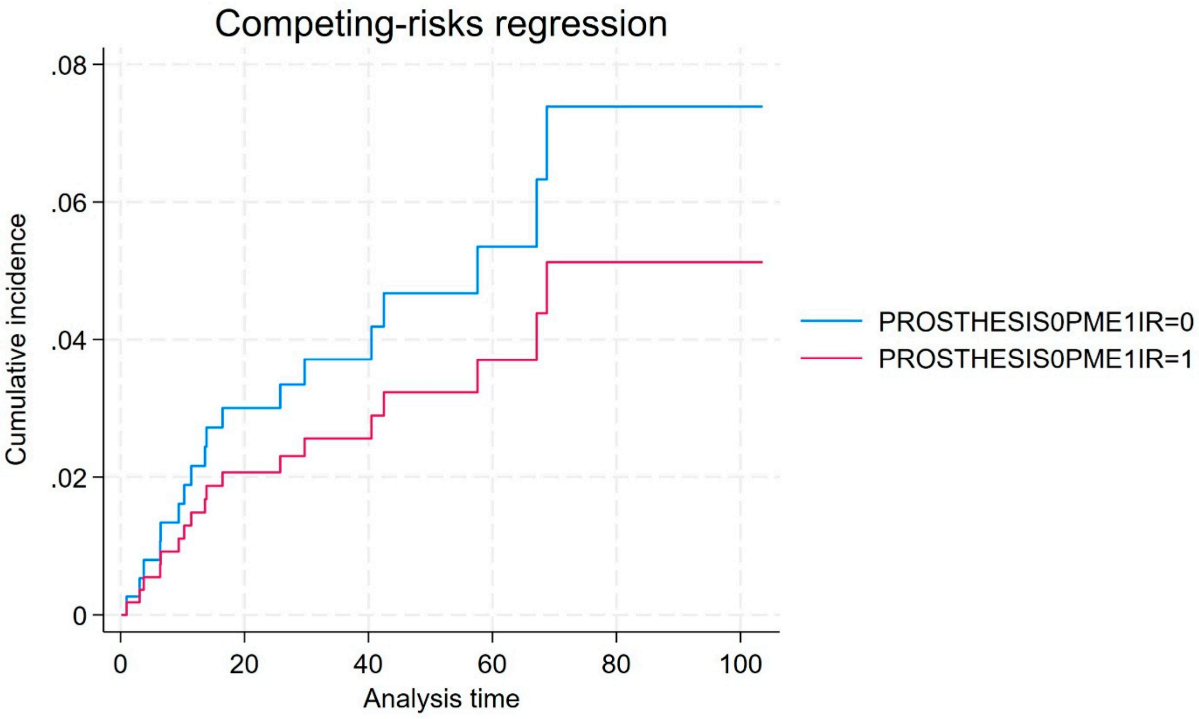

Supplmentary Figure S1. Cumulative incidence function (CIF) of prosthetic valve reintervention in PME and IR groups, with death as a competing risk (Fine and Gray model). No significant difference was observed between groups**.**
